# The Influence of Billboard-Based Tobacco Prevention Posters on Memorization, Attitudes, and Craving: Immersive Virtual Reality Study

**DOI:** 10.2196/49344

**Published:** 2024-07-09

**Authors:** Solenne Bonneterre, Oulmann Zerhouni, Marilisa Boffo

**Affiliations:** 1 Université Paris Nanterre Nanterre France; 2 Université Clermont Auvergne Clermont-Ferrand France; 3 Université Rouen Normandie Rouen France; 4 Erasmus University of Rotterdam Rotterdam Netherlands

**Keywords:** tobacco, smoking, health promotion, health prevention campaigns, immersive virtual reality, incidental exposure, advertising

## Abstract

**Background:**

Health prevention campaigns often face challenges in reaching their target audience and achieving the desired impact on health behaviors. These campaigns, particularly those aimed at reducing tobacco use, require rigorous evaluation methods to assess their effectiveness.

**Objective:**

This study aims to use immersive virtual reality (iVR) to systematically evaluate recall, attitudinal, and craving responses to antitobacco prevention messages when presented in a realistic virtual environment, thereby exploring the potential of iVR as a novel tool to improve the effectiveness of public health campaigns.

**Methods:**

A total of 121 undergraduate students (mean age 19.6, SD 3.7 years), mostly female (n=99, 82.5%), were invited to take a guided walk in the virtual environment, where they were randomly exposed to a different ratio of prevention and general advertising posters (80/20 or 20/80) depending on the experimental condition. Participants’ gaze was tracked throughout the procedure, and outcomes were assessed after the iVR exposure.

**Results:**

Incidental exposure to antitobacco prevention and general advertising posters did not significantly alter attitudes toward tobacco. Memorization of prevention posters was unexpectedly better in the condition where advertising was more frequent (β=–6.15; *P*<.001), and high contrast between poster types led to a better memorization of the less frequent type. Despite a nonsignificant trend, directing attention to prevention posters slightly improved their memorization (β=.02; *P*=.07). In addition, the duration of exposure to prevention posters relative to advertisements negatively affected memorization of advertising posters (β=–2.30; *P*=.01).

**Conclusions:**

Although this study did not find significant changes in attitudes toward tobacco after exposure to prevention campaigns using iVR, the technology does show promise as an evaluation tool. To fully evaluate the use of iVR in public health prevention strategies, future research should examine different types of content, longer exposure durations, and different contexts.

**Trial Registration:**

Open Science Framework E3YK7; https://osf.io/e3yk7

## Introduction

### Background

Tobacco consumption, including both traditional combustible cigarettes and e-cigarettes (vaping), is a major public health concern [1]. Cigarettes are responsible for various health issues such as cancer and cardiovascular diseases [2]. Public health campaigns aim to address the risks associated with both smoking and vaping, with various channels used to broadcast these messages (eg, videos and social media advertising [3]), with billboard prevention posters as a widely used strategy that can display health promotion campaigns in different locations in daily life [4]. However, the effectiveness of these campaigns may vary depending on the type of tobacco product targeted. This study explores the impact of antitobacco prevention messages in a realistic virtual environment (VE), focusing on both vaping and combustible cigarettes. We aim to understand whether there are differences in attitudes and responses to prevention messages based on the type of tobacco product.

### Limitations of Actual Evaluations of Universal Prevention Campaigns

Even if largely used for universal prevention, evidence shows that preventive posters might not be as effective as intended [5]. Several meta-analyses and reviews [3,4,6-9] concluded on a lack of efficacy for most prevention campaigns, which can be explained by unstandardized evaluation, the use of arbitrary outcomes to declare a campaign effective, and sometimes deficits in the design of the campaign itself and its evaluation. Altogether, both laboratory-based and field evaluations of prevention campaigns are considered unsatisfactory [4,7,10], notably because exposure to prevention content in real life is more likely to be incidental (ie, individuals are exposed to prevention messages without being consciously or directly aware of them or without actively processing their content [5,10]) and evaluation methods used so far do not respect this condition of exposure (eg, use of forced exposure paradigms, where individuals are directly exposed to preventive content or stimulus as the primary task). Meanwhile, prevention campaigns lack rigor in their evaluation, as it is difficult to assess to what extent individuals have been exposed to the posters (ie, whether individuals saw or read them), how many times, and so on. Altogether, this points to the need for a systematic and controlled way to recreate ecological situations of exposure to prevention messages, enabling a more effective evaluation of preventive campaigns.

### What Is Incidental Exposure?

Incidental exposure has been mainly explored in the brand sponsorship field [11-14], where individuals are exposed to advertising banners while watching a sporting event. Results from these studies indicate that the more individuals spent time being incidentally exposed to brands, the more positive their attitudes toward the brand, and the more they were able to recall the brand. Similar results were found when recreating incidental exposure to a brand while being exposed to advertising in video games, where advertising was present in the environment participants played in [15].

Concerning tobacco prevention, a few studies have explored the effect of incidental exposure on tobacco evaluation or memorization. Earp et al [16,17] showed that individuals exposed to “no-smoking” signs tended to activate tobacco concepts in memory, leading to more approach tendencies toward tobacco. When facing tobacco prevention in an incidental manner, individuals might process the information but in a less elaborated, biased way [18,19], potentially leading to counterproductive effects (ie, increasing the likeability of tobacco). Two studies used eye-tracking technologies to study incidental exposure to tobacco cues in real life [20,21]. While wearing a portable eye-tracking device, a sample of smokers and nonsmokers were asked to buy either a candy bar or a candy bar and a pack of cigarettes at a real-life store where tobacco advertising and tobacco products were displayed behind the cashier. For participants in the candy bar condition, where no instructions were given about tobacco, recall of either a specific part of the wall displaying information about tobacco products behind the cashier or a promotional offer for tobacco was higher. This implies that even when incidentally exposed to tobacco promotion cues, both smokers’ and nonsmokers’ memory was impacted. Similar results have been found in a virtual environment regarding the processing of tobacco products [22-24].

Altogether, evidence is lacking regarding the effects of prevention posters on memorization and attitudes toward tobacco while being incidentally exposed to tobacco prevention. Moreover, to our knowledge, no study has combined both tobacco prevention messages and general advertising in the same environment—similar to real-life daily environments—to investigate whether being exposed to general advertising will interfere with the processing of tobacco prevention campaigns. As such, this underlines the need to investigate the efficacy of prevention under realistic, incidental exposure recreating an ecological situation where individuals’ attention may be attracted to multiple stimuli (eg, prevention, advertising, surroundings, and distractors). As individuals have limited resources to allocate to their environment [4,19,25], we tested whether this scenario results in a diminished efficacy of prevention content to reach participants’ perception, therefore reducing their impact on attitudes and memorization.

### Immersive Virtual Reality as a Solution to Systematically Evaluate Prevention Campaigns

Immersive virtual reality (iVR) refers to a type of digital technology including a computer-generated VE, in which individuals are immersed and can interact with as they would in real life. iVR usually uses sight as its main sensory channel by using a head-mounted display (HMD) but may also include other sensory feedback (eg, smell and sounds), deepening the immersive experience and enabling interaction with the VE the closest to real life.

The high degree of immersion and presence associated with iVR technology makes it a powerful tool for research to mimic an authentic experience in the laboratory. It is the ideal tool to create an ecological setting in which prevention posters are displayed, including the sources of information and distractions (eg, signs, advertising, and ongoing tasks) that are present in real-life exposure to billboard preventive posters [21]. Additionally, iVR includes eye-tracking measurement through the goggles, which allows us to assess participants’ attention allocated to stimuli of interest (ie, posters) via the time spent looking at them [20,25,26].

### Objectives and Hypotheses

We aimed to explore to what extent being exposed to prevention posters about tobacco in a virtual urban street is effective when in competition with advertising posters displayed in the same VE. We evaluated the impact of incidental exposure to prevention messages on memorization, attitudes, and cravings toward tobacco. We used an 80/20 ratio for the 2 types of posters to expose participants to either mainly prevention or mainly advertising in order to create a clear distinction between the 2 experimental conditions and maximize internal validity (ie, maximizing chances to detect an effect if that exists).

We hypothesized that (hypothesis 1) participants’ attitudes toward tobacco would be more likely impacted (ie, less positive) by prevention posters when those are predominant in the environment and (hypothesis 2) tobacco prevention posters would be better memorized when they are displayed predominantly in the environment compared to when advertisements are predominant. These hypotheses are based on the idea that more repetition of a message would enhance its effectiveness [7,10]. Tobacco cravings were also explored both as an outcome and as a potential predictor of attitudes. In the prevention condition, cravings toward tobacco were expected to be higher than in the advertising condition (hypothesis 3). Variables associated with iVR (namely, presence, immersion, and cybersickness) were explored as covariates for each hypothesis. All hypotheses, procedures, material, and analyses were preregistered.

## Methods

### Participants

We conducted an a priori power analysis [27] assuming a medium effect size (*f*^2^=0.15) for a linear multiple regression (fixed model, *R*^2^ increase with 4 key predictors), which recommended a minimum of 108 participants to ensure 90% power. The mean effect size used in our sample size calculation is based on empirical evidence from previous studies that reported similar effect sizes under analogous conditions. In Zerhouni et al [13], a medium effect size was observed in a study with a comparable intervention and demographics, which is consistent with the scope of our research. Furthermore, Zerhouni et al [14] reinforced these findings in a subsequent study that not only replicated the medium effect size but also extended the research to a similar population, providing additional validation of our assumptions.

Participants were 121 undergraduate students (mean age 19.6, SD 3.7 years; mostly women, n=99, 82.5%) from Université Paris Nanterre, recruited through the web-based research participant recruitment platform for psychology studies. To register, participants needed to complete a preregistration questionnaire containing eligibility screening questions (ie, dichotomic questions asking whether the participant had epilepsy, impaired vision, or memory impairment) and information about the smoking status (detailed below in the “Preregistration Questionnaire” section) alongside a description of the study and the consent form to participate. Participants who did not meet the eligibility criteria (eg, having a normal or corrected vision and no memory impairment) were kindly declined from further subscription to the study. Participants were asked whether they identified themselves as nonsmokers, former smokers, occasional smokers, or regular smokers and the total length of time they had smoked in their lifetime. Most participants were nonsmokers (n=93, 77.5%). Among smoking participants, 9.1% (n=11) were regular smokers, followed by 8.3% (n=10) occasional, and 5% (n=6) former smokers. The data show variability in smoking duration in years within the smoker’s group (mean_occasional_smoker_ 1.98, SD_occasional_smoker_ 1.69 and mean_regular_smoker_ 4.3, SD_regular_smoker_ 4.05), ranging up to 5 years or more, suggesting considerable variation in smoking experience between participants. Despite this variability, test statistics show no significant difference in smoking duration between different smoking statuses (*χ*^2^_2_=35.2; *P*=.85). Scores on the Fagerstrom Test for Nicotine Dependence (FTND) [28] indicated that 66.7% (n=14) of the smokers were nondependent, 23.8% (n=5) lightly dependent, and 9.5% (n=2) moderately dependent. Concerning the use of iVR, 54.2% (n=65) of participants indicated that they already used an iVR device in the past, and only 3.1% (n=2) declared feeling sick when using it.

In total, 60 participants were randomly assigned to the prevention condition (ie, n=10, 80% tobacco prevention posters in the VE) and 61 to the advertising condition (ie, n=10, 80% general advertising posters in the VE). No baseline difference was observed between the groups concerning gender (*P*=.07) and FTND scores (*P*=.17). However, participants in the prevention condition were slightly younger (mean 18.95, SD 1.57 years) than participants in the advertising condition (mean age 20.33, SD 4.89 years; *t*_welch72.6_=–2.09; *P*=.04). Age was then included as a covariate across all analyses.

### Measures and Materials

#### iVR Device, Virtual Environment, and Posters

**Figure 1:**
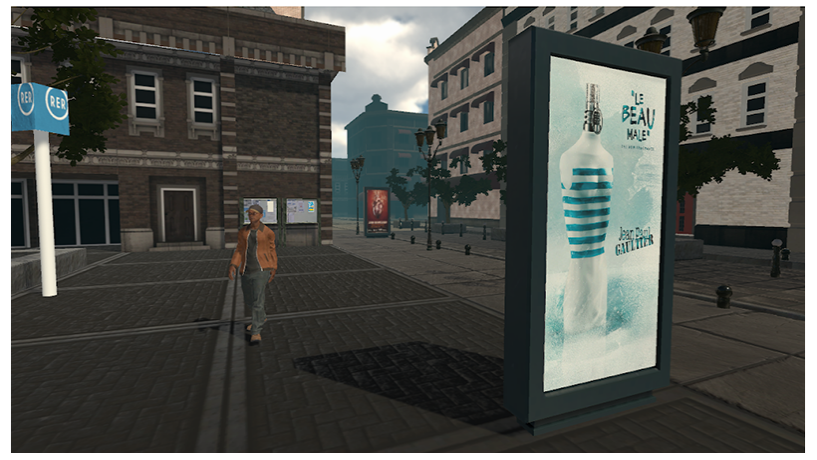
Screenshot of the virtual environment depicting both general advertising and antitobacco preventive posters.

The iVR device was a Sensiks VR pod, in which participants can sit and wear an Oculus Rift 2. Participants moved around the VE using 2 handheld controllers. The VE was designed by the authors in collaboration with a 3D visual design company (Wonderment by Design) and depicted a Parisian suburban area displaying billboards standing on the sidewalk or wall-mounted display panels (Figure 1). Posters were randomly picked from an original pool and randomly displayed depending on the participants’ experimental condition. In both conditions, 15 billboard locations were available, and only 10 of the 15 available billboard locations were randomly picked to display posters. Among the list of 20 possible posters (10 tobacco prevention posters and 10 general advertising posters), 10 were picked randomly (depending on the experimental condition) to be displayed randomly on the 10 selected billboards. The 5 remaining billboards were filled with a blank picture. The randomization of posters and billboard locations was done for every participant. Billboard’s locations in which posters were displayed were randomized among participants. The prevention condition VE included 8 prevention posters and 2 general advertising posters. The advertising condition VE contained 8 advertising posters and 2 prevention posters. Prevention posters (n=10) were real antitobacco prevention posters indicating the health risks of smoking. General advertising posters (n=10) were real advertisements for different commercial products (cars, food, beverage, fragrances, and sports gear), each represented by 2 distinct brands but with similarly looking posters (eg, the 2 car brands depicted a similar type of car, in the same shades of color).

#### Preregistration Questionnaire

Eligibility screening included questions regarding participants’ physical ability to participate and three questions on smoking status: (1) whether the participant used to be a smoker or is currently a smoker, and (2) if so, if they are a regular or occasional smoker, and (3) for how long have they smoked or used to smoke. For smokers, participants completed the FTND [28] to assess the severity of dependence on tobacco. For every item, a number of points are attributed to participants’ answers (from 0 to 3 depending on the item), associated with the severity of the dependence on tobacco, and the total score was calculated to obtain 4 levels of dependence: nondependent (from 0 to 2), low dependent (3 to 4), mildly dependent (5 to 6), and highly dependent (over 7).

#### Attitudes Toward Tobacco

A semantic differential measure assessed attitudes [29] with 4 adjective pairs (risky or safe, not enjoyable or enjoyable, dislike or like, and bad or good) rated on a 7-point scale.

#### Cravings for Tobacco

The French version of the Tobacco Cravings Questionnaire [30] was used. This scale contains 12 items, rated on a Likert scale from 1=strongly disagree to 7=strongly agree, with items such as “I would do almost anything for a cigarette now.” The Tobacco Cravings Questionnaire scale showed high internal consistency within this sample (α=.868; ω=0.896; mean 1.729, SD 1.017).

#### Explicit Recollection of Posters

We measured explicit recognition of both prevention and advertising posters in consecutive order. Participants were first exposed to a list of 30 tobacco prevention posters followed by 10 advertising posters and were asked to indicate whether they had seen them in the VE. Posters used in this study resulted from a web-based survey including 388 participants who rated antitobacco preventive posters based on their perceived persuasiveness and relevance. A score for correct recognition (ie, correctly indicating that a poster was present in the VE) and a score for incorrect recognition (ie, indicating viewing a poster that was not presented or the other way around) was calculated for prevention and advertising posters separately. In total, 2 global scores were computed (difference between correct minus incorrect recognition), 1 for each type of poster (prevention poster recognition and advertising poster recognition). The higher the recognition score, the more correctly posters were recognized (ie, the higher the memorization score).

#### Eye-Tracking Measures

Measures such as gaze duration, fixation count, and saccadic movement toward antitobacco posters are proxies for the depth of cognitive processing and engagement that are integral to the effectiveness of health communication strategies. By quantifying these interactions, we aim to dissect the subtleties of how visual attention correlates with message retention and attitude change, key variables in the field of public health. The choice of these dependent variables aims at bridging the gap between attentional engagement in virtual simulations and potential behavioral outcomes in the real world. Investigating these dynamics in VR could potentially revolutionize our approach to health campaign design, tailoring it to how individuals naturally navigate and process information in their environment. To this end, participants’ gaze was continuously assessed via eye-tracking technology embedded in the HMD while being in the VE. We computed 2 scores: exposure duration and directed gaze. Exposure duration refers to the total duration (in milliseconds) participants had any posters in their field of view, irrespective of the direction of their gaze (ie, the posters could be in the peripheral view for instance). Directed gaze was automatically computed by the eye-tracking system, which calculated the percentage of the area a billboard took up on the screen, transformed it to a score from 0 to 20, and summed it up every 0.2 seconds as long as the poster was still on the screen. Consequently, directed gaze is an indicator of more sustained attention to the posters. For each poster presented in the environment, an exposure duration and a directed gaze score were calculated. Hence, exposure duration is a general indicator of how long a poster was anywhere in the participant’s field of view even when the gaze was not directed at it, whereas directed gaze is the average duration a poster was directly looked at. Exposure duration scores were summed up for each participant within prevention and advertisement posters separately to obtain their total exposure duration. Directed gaze scores were averaged out across prevention and advertisement posters separately. Additionally, a relative score for each eye-tracking measure was calculated by calculating the difference between the respective scores for preventive posters minus the score for advertising posters. The larger the difference, the longer participants were exposed to prevention posters (relative exposure duration) and the more they directly looked at them (relative directed gaze) compared to advertisements.

#### Presence

Presence was assessed using the French version of the iGroup Presence Questionnaire (IPQ) [30]. The IPQ contains 14 questions, with answers to indicate on a scale from –3=not at all to +3=completely agree, such as “Somehow I felt like the virtual world surrounded me.” The IPQ showed excellent reliability in this sample (α=.835; ω=0.849; mean 0.526, SD 0.908).

#### Immersion

Immersion was assessed by asking 2 questions about the iVR device characteristics complementing the IPQ scale: “I was able to move into the virtual environment without thinking about how to use handheld controllers” and “I needed to or should have adjusted the helmet during the procedure.” The questions were answered on a 7-point scale with 1=not at all and 7=completely agree.

#### Cybersickness

We used the Virtual Reality Sickness Questionnaire [31] to measure cybersickness. This 9-item questionnaire asked whether participants felt symptoms of cybersickness (eg, general discomfort and blurred vision) during the VE exposure on a 4-point scale (1=none, 2=slight, 3=moderate, or 4=severe). The cybersickness scale had good reliability (α=.736; ω=.755; mean 1.273, SD 0.330).

### Procedure

The study was described as an experiment on movement and space perception in iVR. We used this cover story to limit demand bias from participants [32] and ensure that exposure to posters was incidental during the procedure. At the end of the experiment, participants were debriefed and informed about the real goals of the study.

On the experimental day, participants came to the laboratory and were randomly assigned to 1 of the 2 conditions. The procedure began with a short tutorial on how to use the iVR device, particularly the 2 handheld controllers to move around the VE. After that, participants wore the HMD and were immersed in the VE. The experimenter started by verifying that participants could use the controllers properly and stayed near the participants to guide them in the VE. Participants were asked to do the following tasks at their own pace: walking to the bus stop, reaching the end of the main street, seeking a red-headed person (ie, virtual agent), and walking the street crossing the VE from right to left, then from the top of the main street to the beginning point. This took about 5 minutes to complete. After that, the experimenter invited the participants to move freely in the VE if they wanted to. Participants then completed all the postintervention measures, starting with measures concerning iVR and the VE (ie, presence, immersion, and cybersickness), then the tobacco attitudes scale, cravings questionnaire, and the poster recognition task.

### Data Analysis

We first screened covariates to evaluate the impact of the iVR on presence, immersion, and cybersickness as an intervention manipulation check. Additionally, a mixed ANOVA was conducted for each eye-tracking measure with poster type as a within-subject factor and group as a between-subject factor to verify that participants saw on average more prevention posters in the prevention condition and more advertisements in the advertising condition (ie, an interaction effect between condition and poster type). In the ANOVA, the untransformed exposure duration scores for the 2 poster types were used.

The main analysis included hierarchical multiple regression models for each main outcome (ie, attitude scores, memorization score for prevention posters, and cravings), controlling first for age, gender, FTND scores, presence, immersion, and cybersickness and successively testing the inclusion of experimental condition, the relative eye-tracking scores, and their interactions with condition as key predictors. The memorization score for advertising posters was added as an additional covariate in the model for the memorization score for prevention posters. Finally, relative exposure duration scores were centered using a *z* score to reduce multicollinearity, which was not necessary for the relative directed gaze scores, as these were already transformed scores (whereas exposure duration was a sum of raw data).

### Ethical Considerations

This study falls under the category of social and human sciences research and does not aim to develop biological or medical knowledge. As such, according to the French Public Health Code (Law 2012-300 of March 5, 2012, known as the Jardé Law), this research did not require the ethical approval of a Committee for the Protection of Individuals. The study was conducted in full compliance with ethical standards and the General Data Protection Regulation. Participation was voluntary, informed consent was obtained from all participants, and the study was designed to ensure anonymity, with no personally identifiable information collected. Prior to participation, all participants were given detailed information about the study and signed an informed consent form to ensure they were fully aware of the nature of the study and their rights. Participants were given the opportunity to ask questions and were informed of their right to withdraw from the study at any time without penalty. To protect participants’ privacy and confidentiality, all data collected during the study were anonymized. Personal identifiers were removed, and data were stored in a secure, password-protected database accessible only to the research team. Participants were compensated for their time with course credits. Compensation was designed to be fair and consistent with the guidelines of the university’s research ethics committee.

## Results

### Experimental Condition Validation

#### Overview

Participants felt moderately present in the VE (mean 0.53, SD 0.91). Immersion was also moderate (mean 4.81, SD 1.29), where the use of the controllers was considered a bit unintuitive (mean 2.97, SD 1.76), whereas the usability of the HMD was highly satisfying (mean 6.21, SD 1.56). Participants did not report cybersickness on average (mean 1.27, SD 0.33). No group difference emerged on any of these variables (see Table 1 for exact *P* values).

The ANOVA for exposure duration did not show any main effect of condition (*F*_1,116_=0.01; *P*=.92) or poster type (*F*_1,116_=0.01; *P*=.93), but it showed a significant interaction between the 2 (*F*_1,116_=1015.33; *P*<.001). Namely, advertising posters were viewed the longer in the advertising condition (mean_ads_ 449,375, SD 11,356 and mean_prev_ 107,395, SD 13,316) and the other way around for the prevention condition (mean_prev_ 449,735, SD 13093 and mean_ads_ 109,738, SD 11,165).

The same appeared for directed gaze, with no main effect of condition (*F*_1,116_=0.26; *P*=.61) nor of poster type (*F*_1,116_=2.52; *P*=.12), while their interaction was significant (*F*_1,116_=4.73; *P*=.03). In the advertising condition, individuals gazed more toward advertising posters (mean_ads_ 9.72, SD 1.51 and mean_prev_ 8.47, SD 2.76) than prevention ones and the other way around in the prevention condition (mean_prev_ 11.95, SD 2.71; mean_ads_ 3.94, SD 1.48).

**Table 1:** Linear regression models.

Predictor variables				β (95% CI)		*t* (*df*=102)		*P* value
**Outcome: attitudes**								
	**Model 1: *R*^2^=0.74; AIC^a^=227; BIC^b^=251; *F*_7,107_=44.1; *P*<.001**							
		Presence	–.10 (–0.24 to 0.03)		–1.48		.14	
		Immersion	.04 (–0.06 to 0.14)		0.84		.40	
		Cybersickness	.01 (–0.37 to 0.38)		0.02		.99	
		Cravings^c^	1.07 (0.91 to 1.22)		13.87		<.001	
		Age	.01 (–0.02 to 0.05)		0.79		.43	
		Gender^c^	.38 (0.05 to 0.72)		2.30		.02	
		FTND^c,d^	–.22 (–0.38 to –0.06)		–2.68		.008	
	**Model 2: *R*^2^=0.74; AIC=236; BIC=274; *F*_12,102_=24.8; *P*<.001 and Δ*R*^2^=0.00; *F*_5,102_=0.17; *P*=.97**							
		Presence	–.11 (–0.25 to 0.03)		–1.51		.13	
		Immersion	.04 (–0.07 to 0.14)		0.67		.50	
		Cybersickness	.02 (–0.37 to 0.42)		0.12		.91	
		Cravings^c^	1.07 (0.91 to 1.23)		13.50		<.001	
		Age	.01 (–0.02 to 0.05)		0.86		.39	
		Gender^c^	.36 (0.01 to 0.71)		2.05		.04	
		FTND^c^	–.22 (–0.39 to –0.06)		–2.69		.01	
		Condition (prevention vs advertising)	.00 (–0.74 to 0.74)		0.00		.99	
		Relative exposure duration	.09 (–0.45 to 0.63)		0.33		.74	
		Relative directed gaze	–.00 (–0.01 to 0.01)		–0.17		.87	
		Condition×relative exposure duration^e^	–.15 (–0.93 to 0.62)		–0.39		.70	
		Condition×relative directed gaze^f^	–.00 (–0.02 to 0.01)		–0.55		.58	
**Outcome: recognition score for prevention posters**								
	**Model 1: *R*^2^=0.15; AIC=572; BIC=594; *F*_6,108_=3.09; *P*=.008**							
		Presence	–.21 (–0.82 to 0.41)		–0.67		.51	
		Immersion	.01 (–0.44 to 0.46)		0.04		.97	
		Cybersickness^c^	1.98 (0.30 to 3.66)		2.34		.02	
		Age^c^	.14 (–0.00 to 0.29)		1.95		.05	
		Gender^c^	1.92 (0.45 to 3.39)		2.59		.01	
		FTND	–.10 (–0.65 to 0.45)		–0.35		.73	
	**Model 2: *R*^2^=0.53; AIC=514; BIC=553; *F*_12,102_=9.74; *P*<.001 and Δ*R*^2^=0.38; *F*_6,102_=14.1; *P*<.001**							
		Presence	–.23 (–0.71 to 0.25)		–0.96		.34	
		Immersion	–.19 (–0.55 to 0.17)		–1.07		.29	
		Cybersickness^c^	1.43 (0.12 to 2.75)		2.17		.03	
		Age	.05 (–0.07 to 0.16)		0.83		.41	
		Gender^c^	1.53 (0.37 to 2.69)		2.62		.01	
		FTND	–.11 (–0.53 to 0.31)		–0.51		.61	
		Recognition advertisement posters	.13 (–0.07 to 0.33)		1.33		.19	
		Condition (prevention vs advertising)^c^	–6.15 (–8.90 to –3.40)		–4.44		<.001	
		Relative exposure duration	.79 (–1.07 to 2.66)		0.84		.40	
		Relative directed gaze	.02 (–0.00 to 0.04)		1.84		.07	
		Condition×relative exposure duration	.40 (–2.23 to 3.04)		0.30		.76	
		Condition×relative directed gaze	.01 (–0.03 to 0.05)		0.44		.67	
**Outcome: cravings**								
	**Model 1: *R*^2^=0.46; AIC=277; BIC=299; *F*_6,108_=15.30; *P*<.001**							
		Presence	.08 (–0.09 to 0.25)		0.95		.34	
		Immersion^c^	–.13 (–0.25 to –0.00)		–1.99		.05	
		Cybersickness	.22 (–0.25 to 0.68)		0.92		.36	
		Age	–.00 (–0.04 to 0.04)		–0.12		.91	
		Gender	.39 (–0.01 to 0.80)		1.92		.06	
		FTND^c^	.67 (0.52 to 0.83)		8.72		<.001	
	**Model 2: *R*^2^=0.47; AIC=285; BIC=321; *F*_11,103_=8.27; *P*<.001 and Δ*R*^2^=0.01; *F*_5,103_=0.38; *P*=.87**							
		Presence	.07 (–0.11 to 0.25)		0.79		.43	
		Immersion^c^	–.13 (–0.26 to –0.00)		–1.99		.05	
		Cybersickness	.20 (–0.29 to 0.68)		0.80		.42	
		Age	–.00 (–0.04 to 0.04)		–0.05		.96	
		Gender	.36 (–0.07 to 0.78)		1.64		.10	
		FTND^c^	.66 (0.51 to 0.82)		8.38		<.001	
		Condition (prevention vs advertising)	–.42 (–1.34 to 0.50)		–0.91		.36	
		Relative exposure duration	.36 (–0.31 to 1.03)		1.06		.29	
		Relative directed gaze	–.00 (–0.01 to 0.01)		–0.49		.63	
		Condition×relative exposure duration	–.34 (–1.30 to 0.62)		–0.71		.48	
		Condition×relative directed gaze	.01 (–0.01 to 0.02)		0.82		.41	

^a^AIC: Akaike information criterion.

^b^BIC: Bayesian information criterion.

^c^Significant predictors.

^d^FTND: Fagerstrom Test for Nicotine Dependence.

^e^Exposure duration: total time in milliseconds the posters were in the field of view.

^f^Directed gaze: averaged running sum of the percentage of area billboards took up of the screen.

#### Hypothesis Testing

Results for all outcomes are displayed in Table 1. We did not find any significant effect concerning attitudes or cravings (see Table 1 for exact *P* values), whereas the inclusion of the key predictors explained an additional 38% of the variance in the memorization of prevention posters measure (Δ*R*^2^=0.38; *F*_6,102_=14.1; *P*<.001). However, contrary to what was expected, participants in the advertising condition better recognized prevention posters than participants in the prevention condition (β=–6.15; *P*<.001). Additionally, relative directed gaze positively predicted recognition of prevention posters (β=.02; *P*=.07), albeit this effect was not statistically significant. Independently of condition, the more participants directly looked at prevention posters relative to advertisements, the better their memorization.

### Post Hoc Exploratory Analysis

As a post hoc analysis, we evaluated whether participants in the advertising condition would also better recognize advertisement posters rather than prevention posters by running the same hierarchical regression model with the recognition score for advertising posters as the outcome (Table 2). The inclusion of the key predictors significantly improved the model which explained an additional 49% of the outcome variance (*P*<.001). Not surprisingly, the longer participants had prevention posters in their field of view relative to advertisements (ie, relative exposure duration), the worse the recognition of advertising posters (β=–2.30; *P*=.01). However, despite being exposed to mostly prevention posters, participants in the prevention condition had significantly higher recognition scores for advertising posters compared to participants in the advertising condition who were actually exposed to mostly advertisements (β=6.63; *P*<.001). This effect appeared to be further moderated by the total duration of exposure to prevention posters relative to advertisements, as shown by the interaction condition×relative exposure duration (β=2.38; *P*=.06), albeit only marginally statistically significant. Compared to the advertising condition, in the prevention condition, the longer participants had prevention posters anywhere in their field of view relative to advertisements, the better their recognition of advertising posters.

**Table 2:** Linear regression model for post hoc analysis.

Predictor variables			β (95% CI)	*t* (*df*=102)	*P* value
**Outcome: recognition score for advertising posters**					
	**Model 1: *R*^2^=0.06; AIC^a^=580; BIC^b^=602; *F*_6,108_=1.17; *P*=.33**				
		Immersion	.15 (–0.31 to 0.61)	0.64	.52
		Cybersickness	–.25 (–1.99 to 1.48)	–0.29	.77
		Age^c^	–.15 (–0.30 to –0.00)	–2.01	.05
		Gender	–.38 (–1.89 to 1.14)	–0.49	.63
		FTND^d^	–.11 (–0.68 to 0.46)	–0.38	.71
	**Model 2: *R*^2^=0.55; AIC=508; BIC=547; *F*_12,102_=10.21; *P*<.001 and Δ*R*^2^=0.49; *F*_6,102_=18.2; *P*<.001**				
		Presence	.34 (–0.12 to 0.81)	1.47	.14
		Immersion^c^	.42 (0.08 to 0.76)	2.44	.02
		Cybersickness	.02 (–1.28 to 1.32)	0.03	.97
		Age	–.07 (–0.18 to 0.04)	–1.19	.24
		Gender	.23 (–0.93 to 1.39)	0.38	.70
		FTND	.00 (–0.41 to 0.42)	0.02	.99
		Memorization prevention posters	.13 (–0.06 to 0.32)	1.33	0.19
		Condition (prevention vs advertising)^c^	6.63 (4.01 to 9.24)	5.03	<.001
		Relative exposure duration^c^	–2.30 (–4.07 to –0.54)	–2.59	.01
		Relative directed gaze	–.01 (–0.03 to 0.01)	–0.80	.43
		Condition×relative exposure duration^e^	2.38 (–0.14 to 4.90)	1.87	.06
		Condition×relative directed gaze^f^	.00 (–0.03 to 0.04)	0.25	.80

^a^AIC: Akaike information criterion.

^b^BIC: Bayesian information criterion.

^c^Significant predictors.

^d^FTND: Fagerstrom Test for Nicotine Dependence.

^e^Exposure duration: total time in milliseconds the posters were in the field of view.

^f^Directed gaze: averaged running sum of the percentage of area billboards took up of the screen.

## Discussion

### Principal Findings

As the literature on incidental exposure and evaluation of preventive campaigns pointed out [3,4,6-9], the use of tobacco prevention posters might not be as effective as previously thought. This study aimed to assess the perception and recall of tobacco prevention posters in a VE containing both advertising and prevention messages. Results indicated that exposure to either prevention or advertising did not significantly alter attitudes toward tobacco, suggesting that either brief incidental exposure is insufficient to influence attitudes or that simultaneous exposure to prevention and advertising may lead to information saturation. Interestingly, in the VE dominated by tobacco prevention content, prevention posters were less memorable than when advertising dominated. Conversely, advertisements were better remembered in a prevention-saturated environment. This counterintuitive finding suggests that content that contrasts with the dominant message type of the environment is more memorable. The findings call for further research on factors that influence the memorization of health prevention messages, such as content tone and relative exposure.

First, we found that attitudes toward tobacco were not significantly different when individuals were exposed to mainly tobacco prevention or mainly advertising in their environment. This might be interpreted in two ways: (1) being incidentally exposed to billboards displaying prevention posters for a few minutes may not be long enough to impact attitudes toward tobacco or (2) being exposed to both prevention and advertising interfere with each other, potentially saturating individuals’ perception or processing of information [18,19], leading to a lack of impact on attitudes toward tobacco. We think that both interpretations are valid and nonmutually exclusive, notably because the literature on the evaluation of universal prevention campaigns pointed out that preventive content is usually unseen or passively walked by [5,10], which results in a lack of efficacy [3,4,6,8]. Moreover, a few studies indicate that promotional content can be more attention-catching than prevention content [20,25], and when exposed incidentally to content, individuals might just preferentially process some information over others [18,19]. Finally, tobacco prevention messages may be perceived as threatening [25]. Therefore, some participants might have just avoided looking at or processing them. Alternatively, habituation to tobacco prevention might occur as individuals get used to being exposed to such messages, and again, they would stop looking at them or processing them [25].

Second, concerning poster memorization, the experimental condition was predictive of memorization of preventive and advertising posters but not as expected. In the VE displaying mainly tobacco prevention content, memorization scores of prevention posters were lower than when mainly advertising posters were displayed. We explored this effect also for the memorization of advertising posters, and the opposite occurred in the condition including mainly advertisements. Further, while controlling for the effect of the other variables, we found that the longer the prevention posters were present anywhere in the participant’s field of view relative to advertisements, the lower the memorization of advertisements; and the more the directed gaze (ie, sustained attention) toward prevention posters relative to advertisements, the better their memorization (although this latter effect just missed the cutoff for statistical significance, *P*>.10 for all).

Altogether, the results seem to indicate that being incidentally exposed to billboard posters in the street may be enough to leave a trace in memory. This is in line with brand sponsorship studies, which found that repetitive incidental exposure to posters enhances memorization [12]. This further highlights the importance of strategically locating billboards and working on their size and look to catch the eye [11,12]. However, the pattern of results related to the memorization of the posters seems to suggest that depending on the proportion of prevention and advertising posters in the environment, the posters presented the least (ie, advertisements in the prevention condition and prevention in the advertising condition) are better recognized, contrary to the expectations. In an environment almost saturated with billboards displaying similar content—as is the case in real life where advertising is dominating our urban environment—a qualitatively and quantitatively different type of content is likely to stand out and be better retained in memory. Of note, prevention posters all highlighted the negative consequences of smoking and may have likely been perceived as very different from the general positive “tone” of advertising. Hence, it is unknown if it is the general negative tone of prevention posters, the relative quantity proportion, or both, that matters in their memorization, calling for further investigation into this effect.

An alternative explanation relates to the experimental setup of our study and the assessment of poster recognition. As participants in the advertising condition were only exposed to 2 preventive posters (and the other way around in the prevention condition), it is quite logical that preventive posters can be better retained in this condition (and vice versa) as the likelihood of having a false recognition is lower. Hence, the amount of posters displayed in the VE is an additional factor to manipulate to clarify these results.

### Limitations

The first limitation of this study is that the sample is composed of students, with only a few smokers mostly nondependent on tobacco. As the focus is on universal primary prevention campaigns (ie, prevention addressed to anyone), having 27 smokers in our sample respond to the prevalence of smokers in real-life conditions [1,33]. In addition, as universal prevention is also designed for nonsmokers to prevent smoking initiation, it is interesting to assess what happens when this population is exposed to preventive poster campaigns to ensure they do not provide counterproductive effects. Only a few male participants were included, and we found that male participants had both better memorization scores for prevention posters (but not for advertising posters) and more positive attitudes toward tobacco. It might be likely that individuals with more positive attitudes toward tobacco have a bias when it comes to retaining tobacco information, such as smokers who tend to give more attention to smoking-related cues [34]. However, it might also be an effect of the low number of male participants; therefore, replication and explicit recruitment of demographic subgroups based on gender and smoking status are needed to assess the robustness of our findings. It is also important to consider the age range of our participants, which was predominantly undergraduate students with a mean age of 19.6 (SD 3.7) years. While the age cohort is relevant for the investigation of attitudes toward tobacco, it must be acknowledged that younger participants, particularly adolescents, may respond differently to prevention messages. Adolescents are generally more susceptible to advertising and may exhibit different patterns of attention and memorization. Future research should aim to replicate this study with a younger demographic. In particular, it would be beneficial to target adolescents. Adolescents represent a vulnerable population, particularly in relation to the formation of attitudes toward tobacco use. This population may be more susceptible to advertising. Therefore, it would be valuable to examine how adolescents respond to prevention messages in immersive virtual environments. Such insights could provide a deeper understanding of the effectiveness of prevention messages and inform the development of more impactful strategies.

It may be possible that the recognition task was maybe not the most relevant to fit experimental needs (ie, a large number of distractors). We suggest assessing both implicit and explicit memorization when studying incidental exposure [35-37]. In addition, even though we found that the condition impacted memorization, this result calls for a replication including a control condition. That way, we could address whether it is the comparison of the ratio between posters or the mere fact of being exposed to posters that impacted memorization in an ecological setting.

A last limitation stands in the external validity of our results: to contrast the 2 conditions, we chose a ratio of 80/20 for the 2 poster types in each condition. However, it is rare, if not nonexistent, to have more prevention posters displayed in real-life urban environments than advertising ones. The ratio of posters is therefore not ecological, but it was a methodological choice to more effectively investigate the impact of prevention posters when displayed with general advertising (ie, maximize internal validity). This study is the first to explore this situation; its aim was to give insights on what is more likely to happen in this kind of environment in a controlled VE setting rather than recreating a real-life ratio. Replication should be conducted by also using multiple ratios (eg, 50/50, 20/80, 10/90, and 5/95) to explore the impact of the ratio while mimicking proportions closer to real life to enhance external validity. In addition, tobacco can be displayed to some extent as a form of advertising content in real life (eg, advertising in vaping stores and movie posters, as can be the case for alcohol [38]), but we chose not to include this kind of advertising in our environment, as explicit smoking advertisements are legally forbidden.

iVR offers a novel approach to evaluating the effectiveness of antitobacco campaigns by simulating the environments in which individuals can make health-related decisions. However, it is important to recognize the limitations associated with the use of this technology. For example, cybersickness is a nontrivial issue that may influence participant responses within the iVR environment and, consequently, their responses to the antitobacco messages, introducing a potential confound. In addition, current technology may not fully capture the visceral and emotional cues that influence behavior in real-world settings. This could potentially limit the extent to which findings can be extrapolated to actual settings in which tobacco-related decisions are made. Participant variability in iVR navigation skills and experience may also introduce additional sources of variability into study results. Familiarity with the technology may affect how individuals interact with the VE and respond to the prevention campaigns, thus affecting the internal and external validity of the study findings. The ecological validity of using iVR to assess campaign impact is another factor to consider. While iVR can create compelling and realistic scenarios, there is an inherent artificiality to virtual experiences. The cognitive and emotional responses elicited in these environments may not fully translate to the complexities of real-world behaviors and attitudes. Indeed, the total exposure to the VE was about 5 minutes, and outcome measures were taken immediately after. An effect on attitudes is likely to need repeated exposure to prevention messages over a longer period to eventually impact the subsequent smoking behavior [4,10]. In addition, participants were sitting during the experiment and moving within the VE using handheld controllers, which might have impacted presence and immersion, therefore also possibly lowering ecological validity.

### Conclusions

To our knowledge, this study is the first to use iVR to evaluate tobacco prevention posters efficacy in an ecological environment (ie, containing both prevention and general advertising). It highlights the role of iVR in enhancing ecological validity and engaging participants in a life-like manner, providing a dynamic methodological alternative to traditional surveys for behavioral research. In addition, our findings challenge conventional health communication strategies by demonstrating that negative antitobacco messages, while more memorable, do not directly influence attitudes, a key determinant of behavior, suggesting the need for innovative approaches to the design and delivery of preventive health campaigns.

Although this study did not find significant changes in attitudes toward tobacco following exposure to prevention campaigns or advertising through iVR, the potential of iVR as an evaluation tool should not be dismissed. Our findings underscore the necessity for further research to elucidate the circumstances under which iVR may be efficacious in modifying attitudes and behaviors. Future studies should consider using a wider range of content, extending the exposure duration, and using a greater variety of contexts in order to fully assess the use of iVR in public health prevention strategies. Altogether, this study indicates that an environment containing both prevention and general advertising messages does not impact individuals’ attitudes toward tobacco as some studies have pointed out [3,4,6-9] while showing a complex pattern of memorization of preventive posters relative to advertisements. Poster memorization does not only depend on the attention given to the posters (eg, gaze allocated to the posters) but on the external environmental context they are embedded in. It is therefore likely that presenting a lower ratio of prevention in an environment saturated with advertising might appear salient by contrast and therefore be better recognized than advertising posters. However, replication is needed to corroborate this result.

## Abbreviations

FTND: Fagerstrom Test for Nicotine Dependence
HMD: head-mounted display
IPQ: iGroup Presence Questionnaire
iVR: immersive virtual reality
VE: virtual environment

## Data Availability

The data sets generated and analyzed during this study can be accessed from the Open Science Framework [39].
